# Chronic exposure to Salton Sea aerosols elicits pulmonary inflammation and shifts in murine lung and fecal microbiome diversity

**DOI:** 10.21203/rs.3.rs-9569949/v1

**Published:** 2026-05-06

**Authors:** Talyssa M. Topacio, Mia R. Maltz, David D. Lo, Marina Zaza, William C. Porter, Linton Freund, Abbey Lyew, David Cocker, Trevor Biddle, Keziyah Yisrael, Diana Del Castillo, Hovanness Dingilian, Ryan W. Drover, Jon Botthoff, Emma Aronson

**Affiliations:** University of California, Riverside; University of Connecticut; University of California, Riverside; University of California, Riverside; University of California, Riverside; University of California, San Diego; University of California, Riverside; University of California, Riverside; University of California, Riverside; University of California, Riverside; University of California, Riverside; University of California, Riverside; University of California, Riverside; University of California, Riverside; University of California, Riverside

**Keywords:** Dust, Salton Sea, aerosol, lung microbiome, gut-lung axis, microbial ecology, environmental chamber, respiratory health, host response, asthma, exposure

## Abstract

Lung disease is rampant around the Salton Sea, California’s largest inland lake and a major source of airborne particulates. To examine root causes of pulmonary disease, we investigated the exposure impacts of spatiotemporal variation in aerosols collected near the Salton Sea on lung and fecal microbiomes. We collected dust during the summer and fall at three different sites around the Salton Sea from 2020 to 2022. Dust was filtered to remove microbial cells and aerosolized for 7-day chronic murine exposures within controlled environmental chambers, after which mouse lung and fecal samples were used for 16S rRNA V3-V4 amplicon sequencing. We verified that chronic exposure to aerosols elicits neutrophilic pulmonary inflammation, particularly in mice exposed to collections from the Wister site near the Salton Sea. We found that spatiotemporal variation drove variation in lung microbiome composition in mice exposed to aerosols from 2022. The lung microbiomes of Salton Sea aerosol-exposed mice were found to increase in alpha-diversity and richness, while simultaneously decreasing in evenness. In contrast, the fecal microbiomes of aerosol-exposed mice decreased in diversity and richness. Our findings suggest that chronic exposure to aerosols from Wister, a site immediately Southeast of the Salton Sea, triggers a systemic stress response in mice characterized by high pulmonary neutrophil recruitment, increased lung microbiome diversity, and decreased fecal microbiome diversity. Back trajectory analyses for aerosol surface type frequencies revealed higher contributions from the Salton Sea in 2020 and 2022 collections from Wister. These findings suggest that chronic exposure to Salton Sea aerosols have impacts on host pulmonary and systemic health, as emphasized by significant but opposing effects on lung and fecal microbiome diversity. Furthermore, these findings demonstrate the variable capacity of environmental aerosol exposure to elicit health consequences relative to seasonal weather events.

## Introduction

We are consistently interacting with environmental microorganisms. Even within the built environment, where infrastructure has been specifically designed to filter air and limit our contact with infectious pathogens, interactions with high-traffic surfaces, plants, animals, and other humans determine which microorganisms we are exposed to on a daily basis ^1^. People are more exposed to airborne particulates and microorganisms in the outside world than within the built environment. The effects of climate change and ecological negligence threaten ecosystem stability and lead to subsequent shifts in microbial communities ^2,3^. As temperatures become more extreme, dust becomes entrained in air currents, and natural resources are spent; novel environmental conditions may result in aeolian microbiomes that threaten to create lasting pulmonary impacts on local community members upon inhalation. ^4–7^

One such environment is California’s Salton Sea basin. This terminal, hypersaline, inland lakebed has been deprived of fresh input from the Colorado River in exchange for a more limited influx of agricultural runoff from the Alamo, New, and Whitewater rivers ^8^. Eutrophication and frequent algal or gypsum blooms increase mortality of local fish and migratory bird populations ^9^. Moreover, rising temperatures in the region have exacerbated the Salton Sea’s receding waterline. As more of the Sea’s lakebed (i.e., playa) is exposed around its perimeter, regional dust events become commonplace, and local residents suffer from disproportionately higher rates of asthma per capita as compared to the total population of California ^2,10,11^.

Our work elucidates the relationship between the basin’s dynamic ecology, local aerosols, and emerging public health outcomes. Sites characterized by seasonal variation in precipitation and wind patterns have been shown to influence local bioaerosol composition, with putative negative effects on the region’s inhabitants. Additionally, temperature and wind-induced biogeochemical cycling in the water column seasonally restructures marine microbial diversity, contributing to variability in sea spray aerosols ^12–14^.

Seasonal variation at the Salton Sea basin alters local dust and seawater microbiomes, coupled with shifts in the biogeochemistry of these substrates. Dust and other aerosol spatiotemporal variation may lead to host pulmonary inflammation, with implications for systemic microbiome responses. As the Salton Sea aerosols vary across sites and season, exposure to these aerosols could play a role in shaping lung microbial diversity and composition, which could consequently alter systemic health or gut microbiomes.

Direct dust exposure may exert a heightened influence on lung microbiome composition as opposed to host-associated factors such as pulmonary inflammation or subsequent changes to lung physiology. In a longitudinal study examining lung microbiome composition and pulmonary disease risk factors, geospatial variables— especially the size of regional particulate matter (PM; i.e., fine PM; PM with an aerodynamic diameter of less than or equal to 2.5 micrometers (PM2.5))— were more influential on patient lung microbiome profiles than individual health or lifestyle variables ^15^. Simulated exposure to aerosols collected near the Salton Sea has been shown to elicit neutrophilic pulmonary inflammatory responses in mice ^16^. Moreover, chronic exposure to Salton Sea aerosols have been shown to change lung microbial communities; these observed changes arose independently of the magnitude of lung neutrophilic recruitment ^17^. Despite lung microbiomes being closely associated with the development and function of the host immune systems ^18^, little is understood about the extent to which systemic pressures influence lung microbial communities. Given the potential for direct impacts of dust exposure on lung microbiomes, we hypothesize that variation in Salton Sea dust composition across sites and seasons will lead to differential impacts on lung microbiome diversity and composition following chronic exposure.

In contrast, the highly metabolic gut microbiome is known to augment proper immune function in the lung via microbially derived signaling molecules along the gut-lung axis ^19,20^. Due to the systemic significance of the gut microbiome and its indirect relationship with inhaled aerosols, we hypothesize that if host exposure to Salton Sea aerosols causes pulmonary inflammatory responses, then those inflammatory responses themselves will primarily alter gut (i.e., fecal) microbiomes to a greater extent than would variation in dust composition.

Ecological changes have been shown to impact public health in diverse contexts, including dramatic shifts in living environments, seasonal temperature fluctuations, and resource availability driven by climate change ^21^. However, the cascading effects of ecological distress on both environmental microbiomes and those of resident communities remain widely uncharacterized. Thus, we explore how site-by-season variation in Salton Sea dust may influence lung and fecal microbiome diversity and composition following chronic exposure.

## Methods

### Salton Sea Dust Collection and Processing

**Figure 1:**
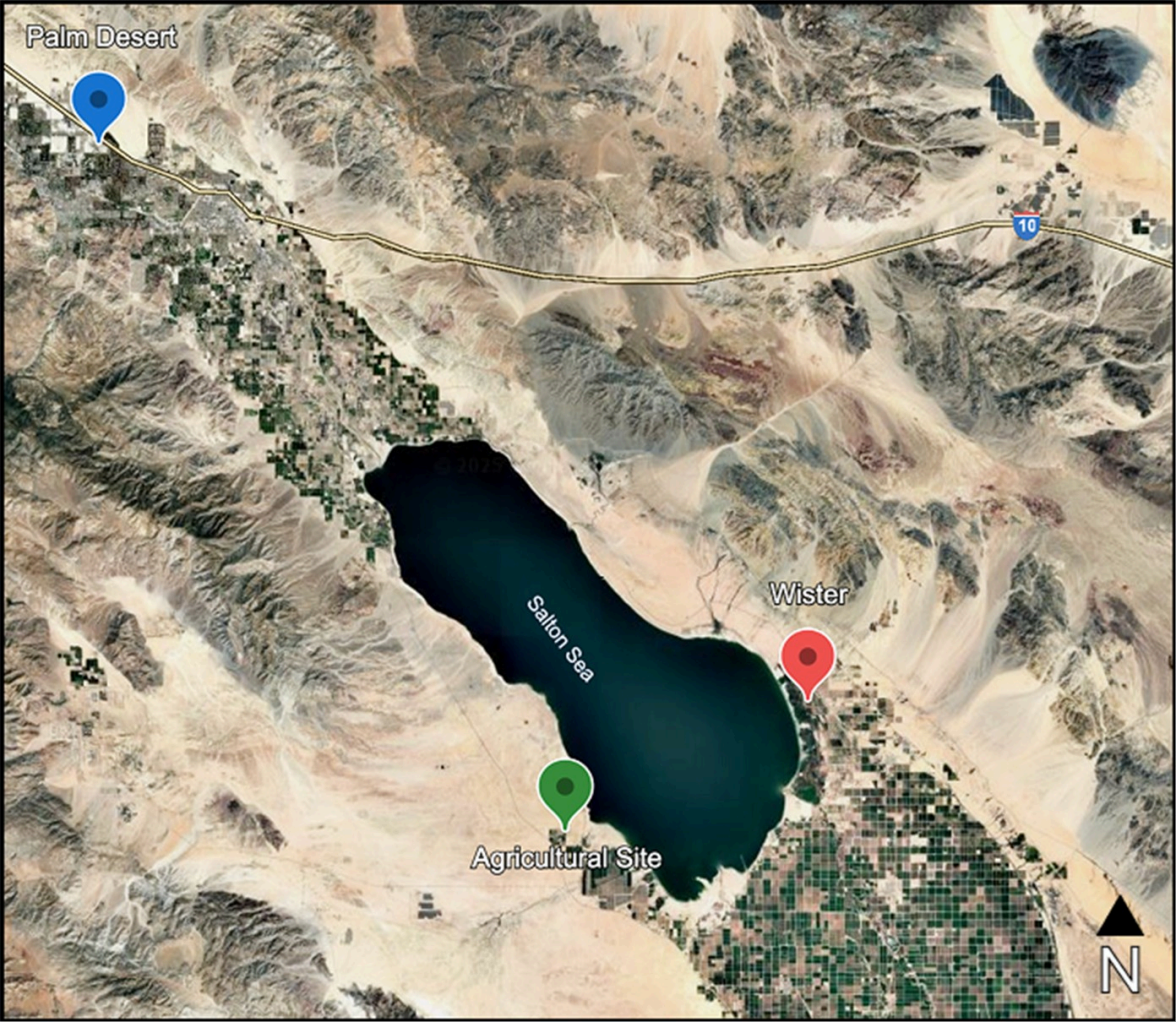
**Map of the Salton Sea** including markers for each dust collection site. Palm Desert (PD, blue) is located approximately 26 miles from the Salton Sea perimeter. Wister (WI, red) and Agricultural site (AG, green) are located <2 miles from the Salton Sea perimeter.

Passive dust collectors ^22^ were deployed at three sites of varying distance to the Salton Sea (Fig. 1) during the fall of 2020, 2021, and 2022. Dust collectors at UC Riverside Palm Desert Campus (PD, 33°46’25.7”N 116°21’10.3”W) were located 25.5 miles northwest from the lakebed’s nearest waterline and were deployed from September 2021-March 2022 and June-September 2022. PD dust exposure previously has been associated with minimal host pulmonary inflammation and minimal influence on mouse lung microbiome composition ^16,17^. Dust collectors at Wister Recreation Area (WI, 33°17’01.9”N 115°36’00.3”W) were located less than 2 miles off the southeastern edge of the Salton Sea and were deployed from August-October 2020, September-December 2021, and June-September 2022. Exposure to WI dust has significantly increased host neutrophil recruitment and influenced the composition of the mouse lung microbiome. Dust collectors at the Agricultural site (AG, 33°10’07.9”N 115°51’21.8”W) were located less than 2 miles off the southwestern edge of the Salton Sea. Aside from being a working agricultural site, AG was selected because of its distance from the sea and placement along its perimeter. These collectors were deployed from September-December 2021 and June-September 2022.

Following the deployment period, dust collectors were rinsed in 1L MilliQ water. The resulting aqueous suspensions were vacuum filtered through a 0.2 μM mesh to allow water-soluble components and minerals to pass through while blocking viable, intact microbial cells and large particles. The remaining filtrate was frozen at −20°C for 24–48 hrs and lyophilized to concentrate (Labconco, Kansas City, MO), then resuspended in MilliQ water before aerosolization.

### Animal Use Ethics

All work involving the use of live mice was done in compliance with the University of California, Riverside’s Institutional Animal Care and Use Committee (IACUC) and National Institute of Health (NIH) guidelines. Male and female C57BL/6 mice were purchased from Jackson Labs (Sacramento, CA) at 8 weeks old. Mice were acclimated for approximately one week in a specific-pathogen free vivarium (University of California, Riverside) before use in our chamber exposure studies.

### Chamber Exposures

Cages of 3–4 C54BL/6 mice were randomized between control air and aerosol exposure treatment chambers ^23^ with access to food and water. Equal distribution of male and female mice was maintained between both chambers.

For the duration of each 7-day experiment, the control chamber was filled with dry, filtered air. In the aerosol exposure chamber, aqueous dust suspension was aerosolized with filtered air and passed through a silica drying column ^24^. The aerosolized filtrate was maintained at an average of 1500 μg/m^3^ over the 7-day exposure.

### Mouse Lung & Fecal Dissection

At the end of each exposure period, mice were euthanized with isoflurane and cervical dislocation as per humane animal use protocols. The lower respiratory tract was extracted from below the mid-trachea, then floated and washed in 1x phosphate buffered saline (PBS) before the left and right lung lobes were separated at the tracheal bifurcation. For a subset of experimental mouse cohorts (PD 2022, AG 2022, and WI 2022), individual fecal pellet samples were collected directly from the large intestine. Lung and fecal samples were stored in their respective collection tubes at −80°C before microbial DNA extraction.

### Flow Cytometry

Bronchoalveolar lavage fluid (BALF, 2.4mL) was collected from a subset of mice for digestion and staining for flow cytometry. Samples were stained with fluorescent antibodies: anti-CD45 FITC (BioLegend, San Diego, USA; Clone 30-F11), anti-CD19 PE-Cy5 (eBioscience, San Diego, USA; Clone MB19–1), anti-CD3 Alexa Fluor 700 (BioLegend, San Diego, USA; Clone 17A2), anti-Ly6G BV510 (BioLegend, San Diego, USA; Clone 1A8), anti-CD11b BV421 (BioLegend, San Diego, USA; Clone M1/70), anti-CD11c PE-Cy7 (BioLegend, San Diego, USA; Clone N418) and anti-SiglecF APC (BioLegend, San Diego, USA; Clone S17007L).

Flow cytometry was performed on a Novocyte Quanteon (Agilent, Santa Clara, CA), and gating was done using FlowJo (Version 10.71). Cell count data was analyzed in RStudio (R software version 3.18). The Shapiro-Wilks test was used to determine a non-normal distribution of neutrophil recruitment data among all BALF samples (P < 0.001), so pairwise comparisons between contemporaneous control air and aerosol-exposure groups were performed with a Wilcoxon signed-rank test.

### Lung Microbiome Library Prep and Sequencing

Whole- or half-lung lobes were extracted using a modified protocol for the HostZERO Microbial DNA extraction kit (Zymo Research, Irvine, CA) for solid tissue samples ^17^. This modification included tissue lysis on an MP Bio Fastprep Classic (MP Biomedicals, Irvine, CA) with 2.0mm beads. During the host depletion protocol, microbial components were separated from eukaryotic cells by centrifugation, then incubated with proteinase K, and mechanically lysed and centrifuged before proceeding with the manufacturer’s recommended protocol. Final sample yield was maximized by duplicating intermediate supernatant collections, which were combined as a single sample (per animal) at the end of the protocol. Negative controls were included in each DNA extraction and used in downstream analyses to control for contaminants. Samples were quantified with a dsDNA High Sensitivity Qubit assay kit (Invitrogen, Carlsbad, CA), and those with detectable concentrations of DNA were sent to Zymo Research (Irvine, CA) for 16S V3-V4 rRNA targeted-amplicon library preparation and gene sequencing.

Samples were treated with a PCR inhibitor removal step using the OneStep PCR Inhibitor Removal Kit (Zymo Research, Irvine, CA). Next, 16S rRNA V3-V4 amplification was done using the Quick-16S NGS Library Prep Kit (Zymo Research, Irvine, CA) with added mitochondrial-specific Peptide Nucleic Acid (mPNAs; mitochondrial blockers) clamps to minimize amplification of eukaryotic host mitochondrial DNA ^25^. Library sequencing was done on an Illumina NextSeq2000^™^ using the P1 reagent kit (600 cycles) and a 30% PhiX spike to promote read diversity.

### Fecal Microbiome Library Prep and Sequencing

One fecal pellet per mouse was extracted for microbial DNA using the Qiagen PowerSoil Pro kit (Qiagen USA, Germantown, MD) as per manufacturer’s protocols with exception to a bead beating and heated lysis step. Samples were homogenized in the bead beater in 1-minute increments for 5 minutes total (MP Fastprep-24, MP Biomedicals, Irvine, CA) before an overnight heated lysis at 65°C. After proceeding with the kit protocol, samples were eluted into 50uL of elution buffer.

DNA extracts were amplified using Klindworth 16S rRNA V3-V4 forward and reverse primers ^26^ and KAPA HiFi HotStart ReadyMix (Roche Molecular Systems Inc., U.S.A.). For amplification PCR, samples were denatured at 95°C for 3 minutes, then 35 cycles were repeated for the following steps: 95°C for 30 seconds, 55°C for 30 seconds, and 75°C for 30 seconds. This was followed by a final extension at 72°C for 5 minutes before samples were stored at 4°C. PCR products were cleaned with AMPure XP beads (1:0.8 ratio, Beckman Coulter Life Sciences Inc., U.S.A.) before indexing. Indexing was done using Illumina Nextera XT indexing primers (Illumina, San Diego, CA) and KAPA HiFi HotStart ReadyMix, with the same thermal cycler settings repeated with 8 cycles instead of 34. Indexed products were cleaned with AMPure XP beads (1:1). Products were quantified with a Qubit dsDNA High Sensitivity assay kit (Invitrogen, Carlsbad, CA) before pooling for sequencing. Amplicon sequencing was done with Illumina Miseq 2×300 paired end reads at University of California, Riverside’s Genomics Core (Riverside, CA).

### Bioinformatics– 16S rRNA Amplicon Sequence Analysis

All 16S rRNA (V3-V4) amplicon sequence data were analyzed using a DADA2 workflow developed by Freund ^12^. Sequence quality was assessed using FastQC and eestats2 ^27^. Amplicon sequence variants (ASVs) were assigned in DADA2 ^28^ using the Silva Database v138.2 ^29^. Negative controls were used to identify potential contaminants in their respective library and were removed using the R “decontam” package ^30^.

### Statistical Analysis

All statistical analyses for 16S rRNA amplicon sequencing data were performed in RStudio (R software version 3.18). Taxa data from multiple Illumina MiSeq 16S rRNA amplicon sequencing runs were merged at the genus-species level before diversity analyses. Alpha-diversity analyses were conducted using the R “vegan” package, and the Shapiro-Wilks test was used to determine distribution normality. For non-normal data, pairwise comparisons between contemporaneous exposure groups and between same-site dust-exposure groups were performed using a Wilcoxon signed-rank test. For normally distributed data, a two-sample t-test was used.

The R “vegan” package was used for beta diversity analyses. Species count data was transformed by center-log ratio (CLR, R “decostand” function) to generate an Aitchison distance matrix ^31–33^ and visualized with a Principal Coordinates Analysis (PCoA). Homogeneity of variance was analyzed using “betadisper” function and permutational multivariate analysis of variance (PERMANOVA) was performed using “adonis2” function, followed by pairwise comparisons with “pairwiseAdonis” function ^34^.

### Limulus Assay for Endotoxin Quantification of Dust

Aqueous Salton Sea dust suspensions were dehydrated prior to measuring average dust mass per collection site using a microbalance (Mettler-Toledo, Columbus, OH). We quantified lipopolysaccharide (LPS) concentrations using the GenScript ToxinSensor Chromogenic LAL Endotoxin Assay kit (Piscataway, NJ, U.S.A.) according to manufacturer’s protocols with a standard curve from 0–1 endotoxin units (EU). Dry, lyophilized dust was resuspended in 100uL of Limulus assay (LAL) reagent water. A maximum volume dilution (MVD) of 0.1EU/mL was calculated based off kit standards, and 100uL total volume was used per sample for the assay according to the manufacturer protocol. Average dust mass was used to calculate average EU/g of LPS per site.

### Dust Surface Type Frequencies

Hourly back trajectory data from the National Oceanic and Atmospheric Administration’s Hybrid Single Particle Lagrangian Integrated Trajectory (HYSPLIT) dispersion and trajectory model ^35^ were used in tangent to data collected from nearby EPA (Environmental Protection Agency) surface stations to determine likely surface sources of particulate matter by site and collection period. Surface type frequencies were calculated using back trajectory endpoints weighted by wind speed, distance, and sediment availability to model source location differences between sites. following the procedure described in Miao et al.^14^. Corresponding surface categories by site were then determined according to the National Land Cover Database (NLCD) classes, including developed land, barren land, forest, crop, herbaceous, shrub, and ocean surface types. The Salton Sea was added as a unique surface category to represent possible contributions from its surrounding playa and from lake spray coming directly from its surface.

## Results

### Lung Microbiome Composition and Dust Collection Season

**Figure 2:**
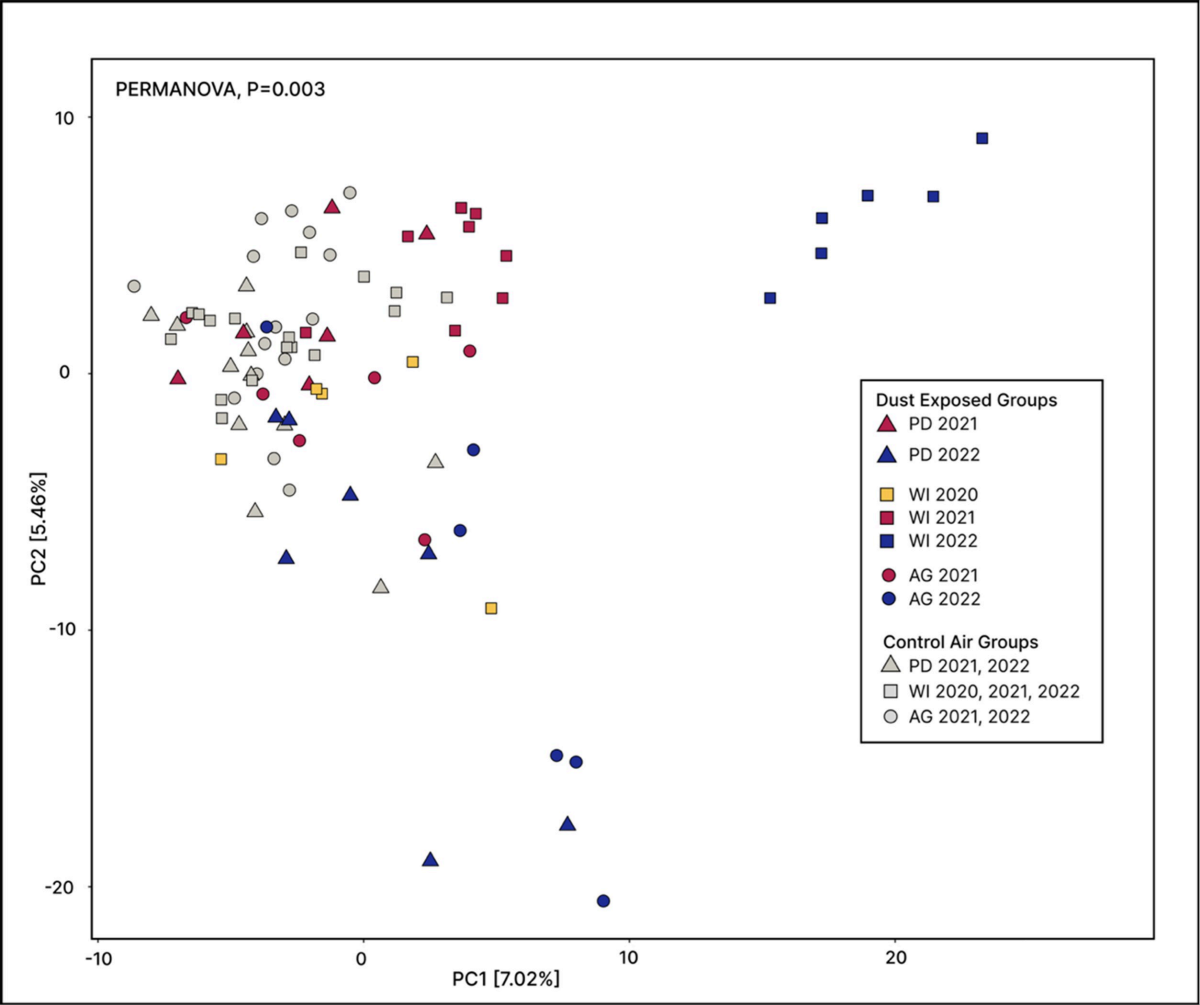
Lung microbiome beta-diversity. Principal coordinates analysis (PCoA) on mouse lung microbiomes using center-log ratio transformed distances (CLR Aitchison). Points are shaped according to dust collection site and colored by dust collection year. Variation among exposure material groups was significantly different (PERMANOVA, P=0.003).

We used a Principal Coordinate Analysis (PCoA) based on Aitchison distance matrices to visualize compositional differences in mouse lung microbiomes according to exposure material (Fig. 2). An analysis of variance (ANOVA) test showed that dispersion among exposure material groups were not significantly different (P = 0.2627). Yet, lung microbiome composition varied significantly by exposure material (P = 0.001). Pairwise comparisons revealed that the lung microbiomes of control air-exposed mice differed significantly from WI 2022 (P < 0.001), AG 2022 (P < 0.001), and PD 2022 (P < 0.001) dust-exposed mice. In addition, lung microbiome composition of WI 2022 dust-exposed mice differed significantly from that of WI 2021 dust-exposed mice (P < 0.001).

### Lung Microbiome Diversity by Exposure Material

**Figure 3:**
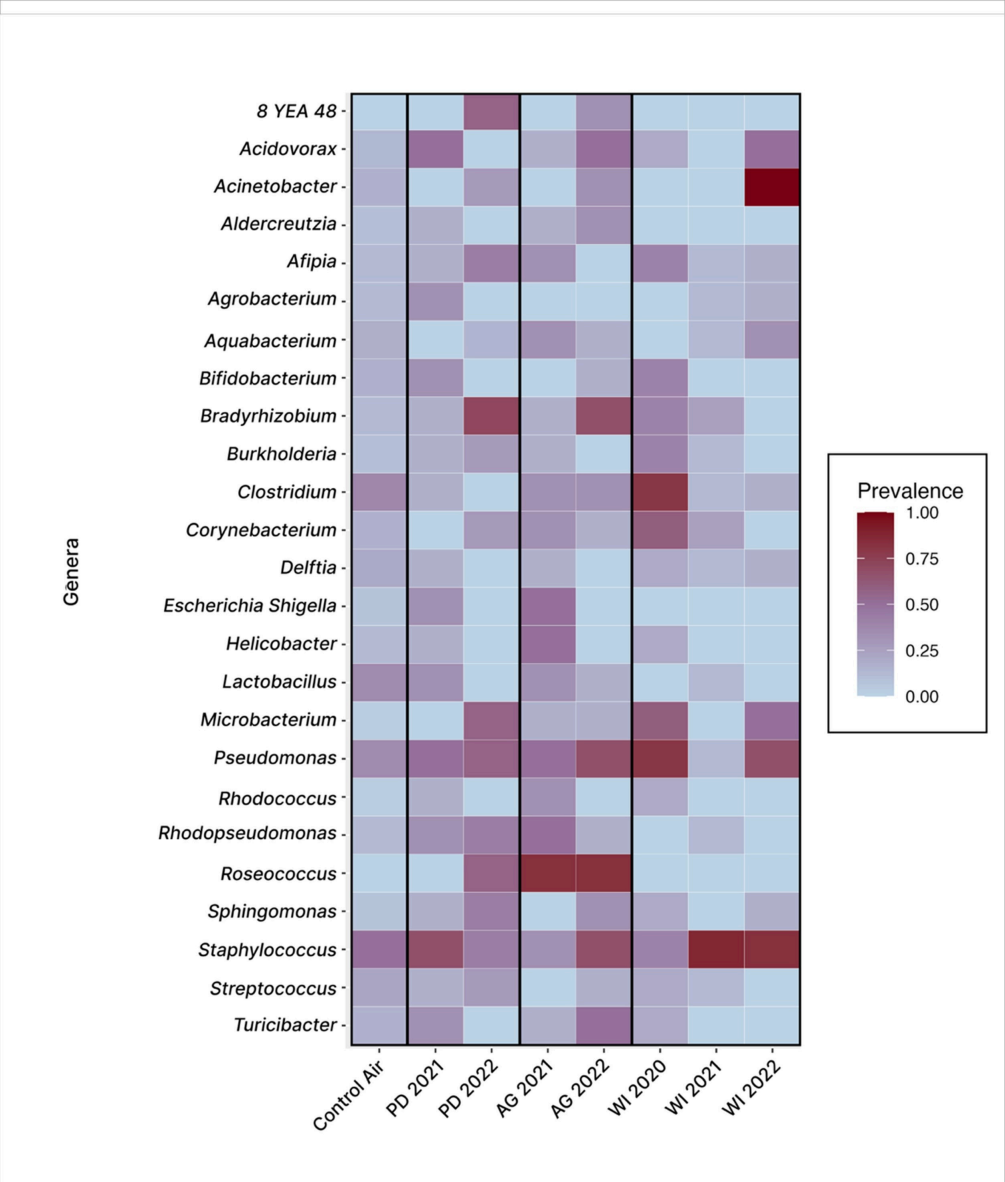
Top 25 genera prevalence by exposure material. Heatmap depicting prevalence (presence in samples at 1% relative abundance) highlights variation in evenness among samples according to exposure material.

The core microbiome analysis by Lahti and Shetty ^36^ was used to visualize evenness and prevalence in mouse lung microbiomes according to exposure material. Prevalence was calculated for samples within each exposure material group based on a 1% genera relative abundance threshold; the top 25 genera across all samples were used to generate a heat map (Fig. 3), along with a table consisting of the top 100 genera (Table S1). A Shapiro-Wilk’s test determined that evenness was not normally distributed (P < 0.001); therefore, a Kruskal-Wallis test was used to reveal that Pielou’s evenness varied significantly among groups according to exposure material (P = 0.003). We used Dunn’s post-hoc test to determine that lung microbiome evenness in WI 2020 dust-exposed mice was significantly higher than that of WI 2021 dust-exposed mice (P = 0.013) and WI 2022 dust-exposed mice (P = 0.032). A significant difference in evenness was also observed between lung microbiomes of WI 2020 dust-exposed mice and AG 2022 dust-exposed mice (P = 0.048).

Clinically relevant taxa including *Acinetobacter, Staphylococcus*, and *Pseydomonas* were enriched among dust-exposed groups. *Acinetobacter* was noticeably enriched in all 2022 dust-exposed groups, *Staphylococcus* was prevalent across sample groups but particularly enriched in WI 2021, WI 2022, AG 2022, and PD 2021 dust-exposed samples, and *Pseudomonas* was more prevalent among all 2022 and WI 2020 dust-exposed group.

**Figure 4:**
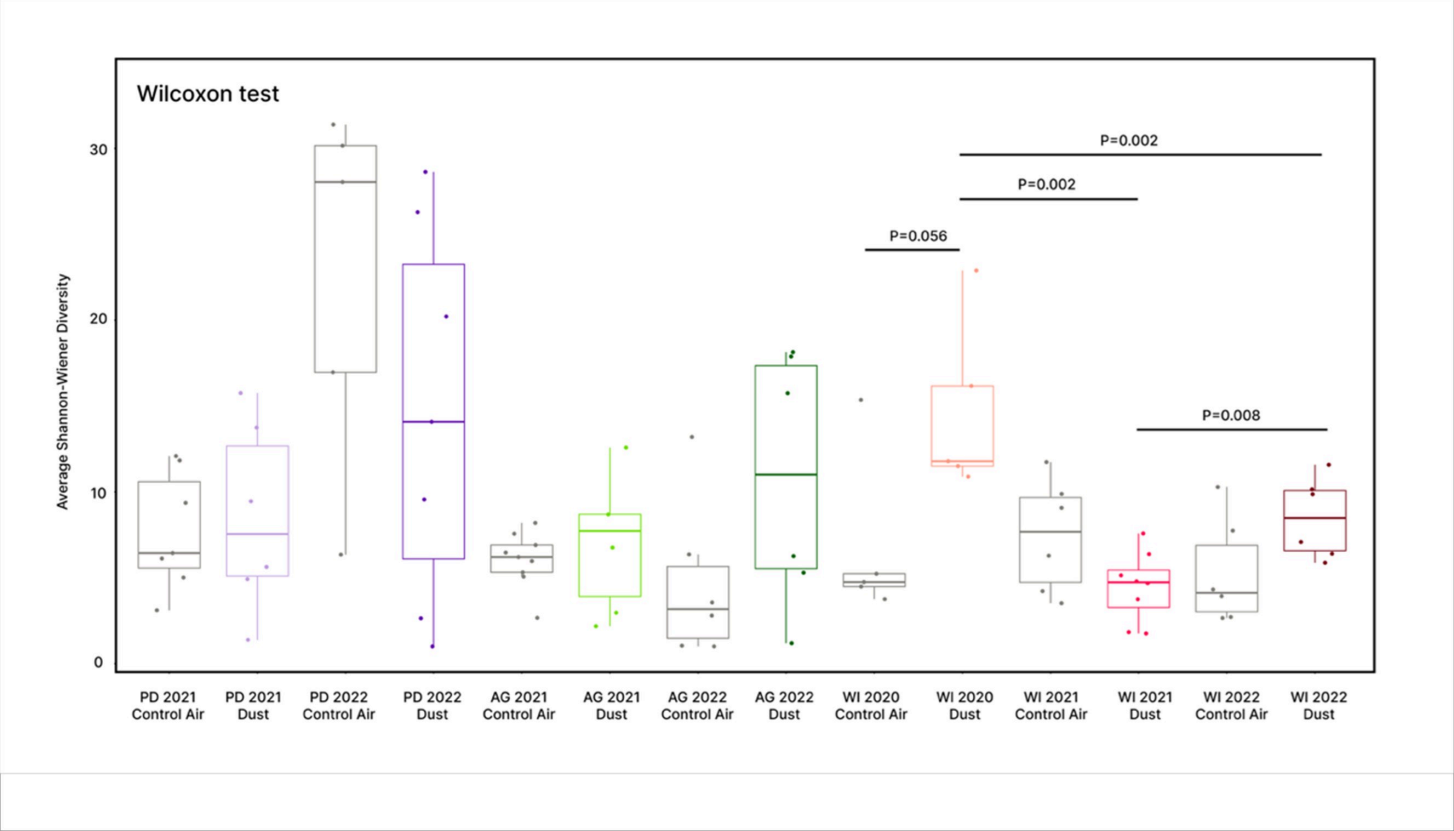
Lung microbiome Shannon-Wiener diversity. Average Shannon-Wiener diversity indexes were plotted according to treatment group and a Wilcoxon test was used to determine significant differences between contemporaneous or same-site pairs. Lung microbiome diversity in WI 2020 dust-exposed mice was significantly different from that of WI 2021 dust-exposed mice (P=0.002) and WI 2022 dust-exposed mice (P=0.002). Lung microbiome diversity in WI 2021 dust-exposed mice were also significantly different from WI 2022 dust-exposed mice (P=0.008).

**Figure 5:**
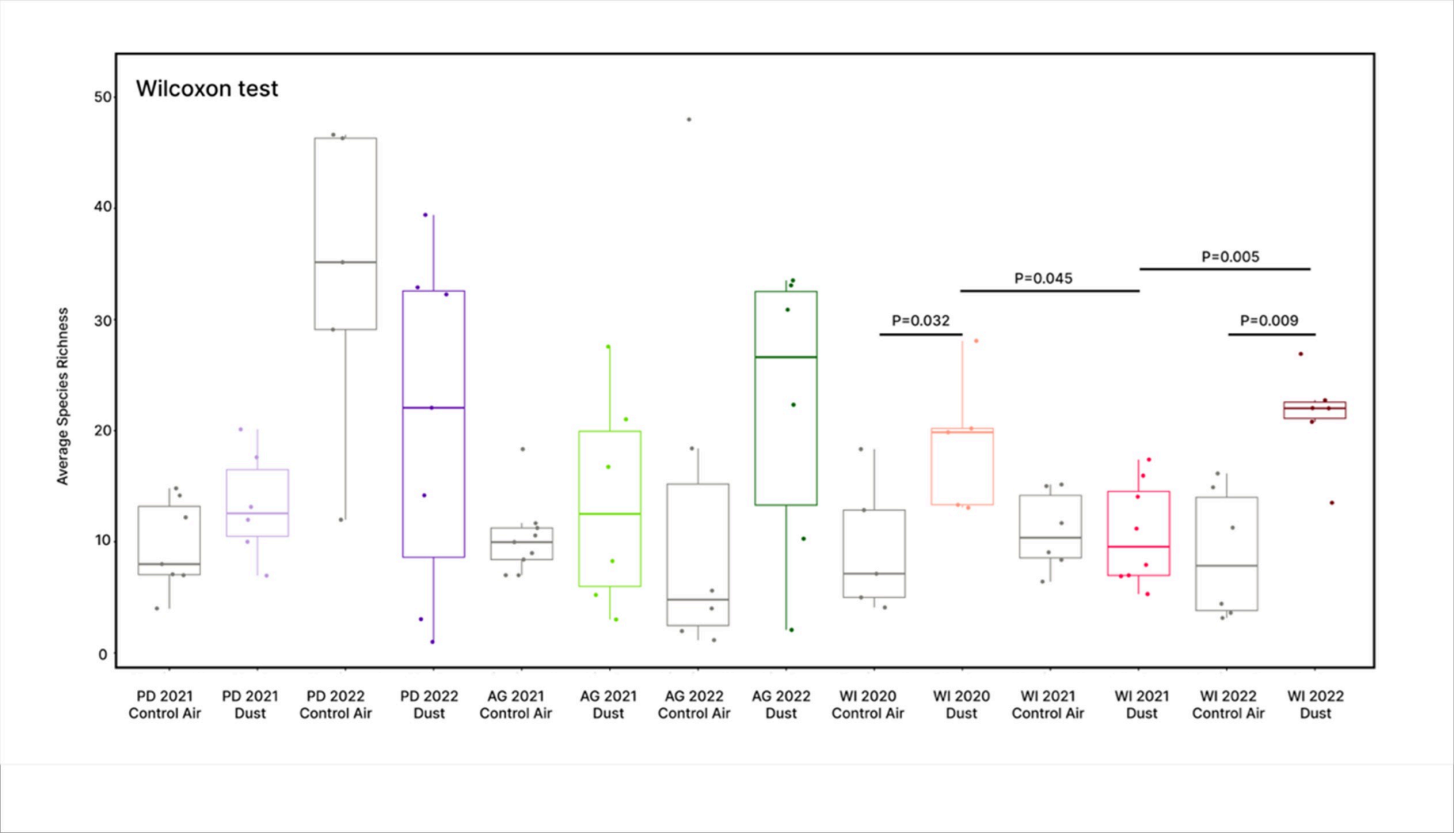
Lung microbiome species richness. Average species richness was plotted according to treatment group and a Wilcoxon test was used to determine significant differences between contemporaneous or same-site pairs. Compared with their contemporaneous control air-exposed groups, lung microbiome richness differed significantly in WI 2020 dust-exposed mice (P=0.032) and WI 2022 dust-exposed mice (P=0.009). Lung microbiome richness in WI 2021 dust-exposed mice significantly differed from WI 2020 dust-exposed mice (P=0.045) and WI 2022 dust-exposed mice (P=0.009).

We calculated Shannon-Wiener diversity and species richness for each treatment group. The Shapiro-Wilk test found that both Shannon-Wiener diversity and species richness were non-normally distributed (Shannon-Wiener, P < 0.001; species richness, P < 0.001), so a Wilcoxon signed rank test was used to analyze pairwise differences in means between contemporaneous treatment groups and same-site dust-exposure groups. For Shannon-Wiener diversity (Fig. 4), no contemporaneous pairs differed significantly from each other; however, lung microbiome diversity of mice exposed to WI 2021 dust differed significantly from that of mice exposed to WI 2020 dust (P = 0.002) and WI 2022 dust (P = 0.007). The lung microbiome diversity of mice exposed to WI 2020 dust also differed significantly from mice exposed to WI 2022 dust (P = 0.002). For species richness (Fig. 5), lung microbiomes from WI 2020 dust-exposed mice differed significantly from their contemporaneous control-air group (P = 0.032). This was observed similarly among WI 2022 dust-exposed mice and their respective contemporaneous control group (P = 0.009). Species richness in WI 2021 dust-exposed lungs differed significantly from WI 2022 dust-exposed lungs (P = 0.005) and marginally differed significantly in comparison to WI 2020 dust-exposed lungs (P = 0.045).

### Fecal Microbiome Diversity and Composition After Dust Exposure

**Figure 6:**
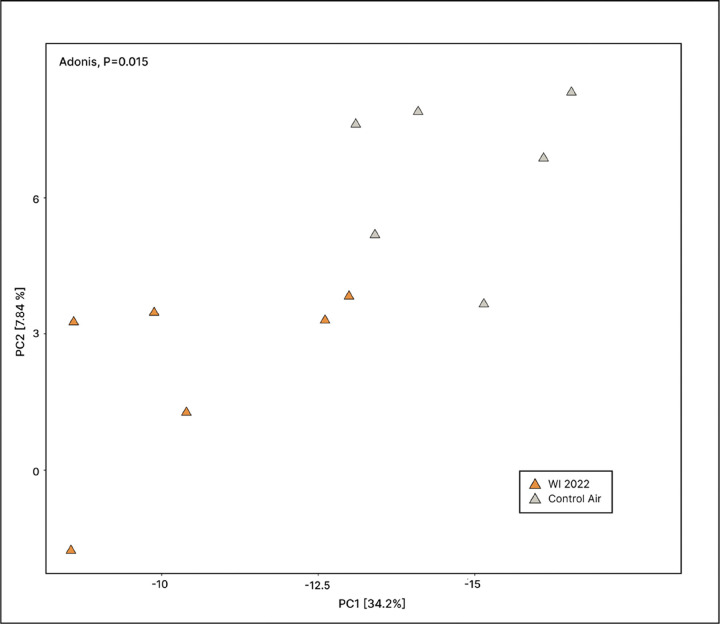
Fecal microbiome composition in WI 2022 dust-exposed mice. A principal coordinate analysis (PCoA) using center-log ratio (CLR, Aitchison) distances revealed dissimilarities in fecal microbiome composition for WI 2022 dust-exposed mice and their contemporaneous controls. Pairwise Adonis revealed this difference to be significant (P=0.015).

Variation in fecal microbiome composition of mice exposed to 2022 dust was visualized using PCoA with Aitchison distances. A PERMANOVA determined that this variance differed significantly (P = 0.003); however, distinct cohort biases among treatment groups can be observed in the PCoA (Fig. S2). A post-hoc test (“pairwiseAdonis”) was used to determine variation between contemporaneous treatment groups and revealed a significant difference between the fecal microbiome composition of WI 2022 dust-exposed mice and their contemporaneous controls (Fig. 6, P = 0.015). No additional significant pairwise comparisons were observed between contemporaneous groups.

**Figure 7:**
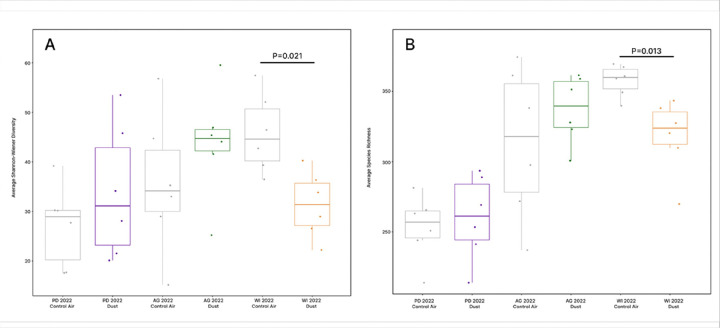
Mouse fecal microbiome diversity according to treatment group. **(A)** Shannon-Wiener diversity was compared between contemporaneous pairs using a two-sample t-test and revealed fecal diversity in WI 2022 dust-exposed mice to be significantly lower than their respective control air-exposed group (P=0.021). **(B)** Species richness was similarly compared using a Wilcoxon signed rank test and revealed fecal species richness in WI 2022 dust-exposed was significantly lower than their control air exposed group (P=0.013).

To minimize the influence of cohort-attributed biases between experimental groups, we used pairwise tests to compare Shannon-Wiener diversity and species richness only between dust-exposure groups and their respective control-air groups. The Shapiro-Wilk test found that Shannon-Wiener diversity data for mouse fecal microbiomes were normally distributed across all samples (P = 0.63), while species richness was non-normally distributed (P = 0.03). A two-sample t-test found that Shannon-Wiener diversity differed significantly in fecal microbiomes of WI 2022 dust-exposed mice when compared to their contemporaneous controls (Fig. 7A, P = 0.021). Similarly, a Wilcoxon signed rank test determined that species richness differed significantly in fecal microbiomes of WI 2022 dust-exposed mice as compared to their contemporaneous controls (Fig. 7B, P = 0.013). In contrast, Shannon-Wiener diversity and species richness were not significantly different for fecal microbiomes of PD 2022 dust- or AG 2022 dust-exposed mice and their contemporaneous controls.

### Compositional Variation Between Lung and Fecal Microbiomes

**Figure 8:**
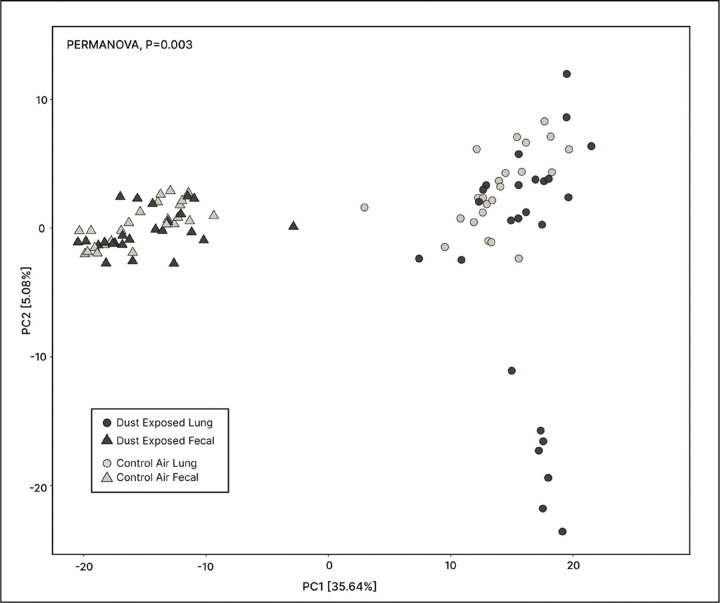
Comparing composition of mouse lung and fecal microbiomes. A principal coordinates analysis (PCoA) was used to visualize the composition of lung and fecal microbiomes, which were significantly distinct from each other, regardless of dust or control air treatment (PERMANOVA, P=0.003).

Lung and fecal microbiome data from PD 2022, AG 2022, and WI 2022 exposures were examined based on chamber (Control vs. Dust) and sample type (Fecal vs. Lung) and subsequently visualized in a PCoA using an Aitchison distance matrix (Fig. 8). We revealed that lung and fecal microbiomes remained distinct from each other; this distinction was confirmed via PERMANOVA, which illustrated significant differences in variation between lung and fecal microbiome composition according to chamber and sample type (P = 0.003).

### Host Inflammation in Response to Salton Sea Dust Exposure

**Figure 9:**
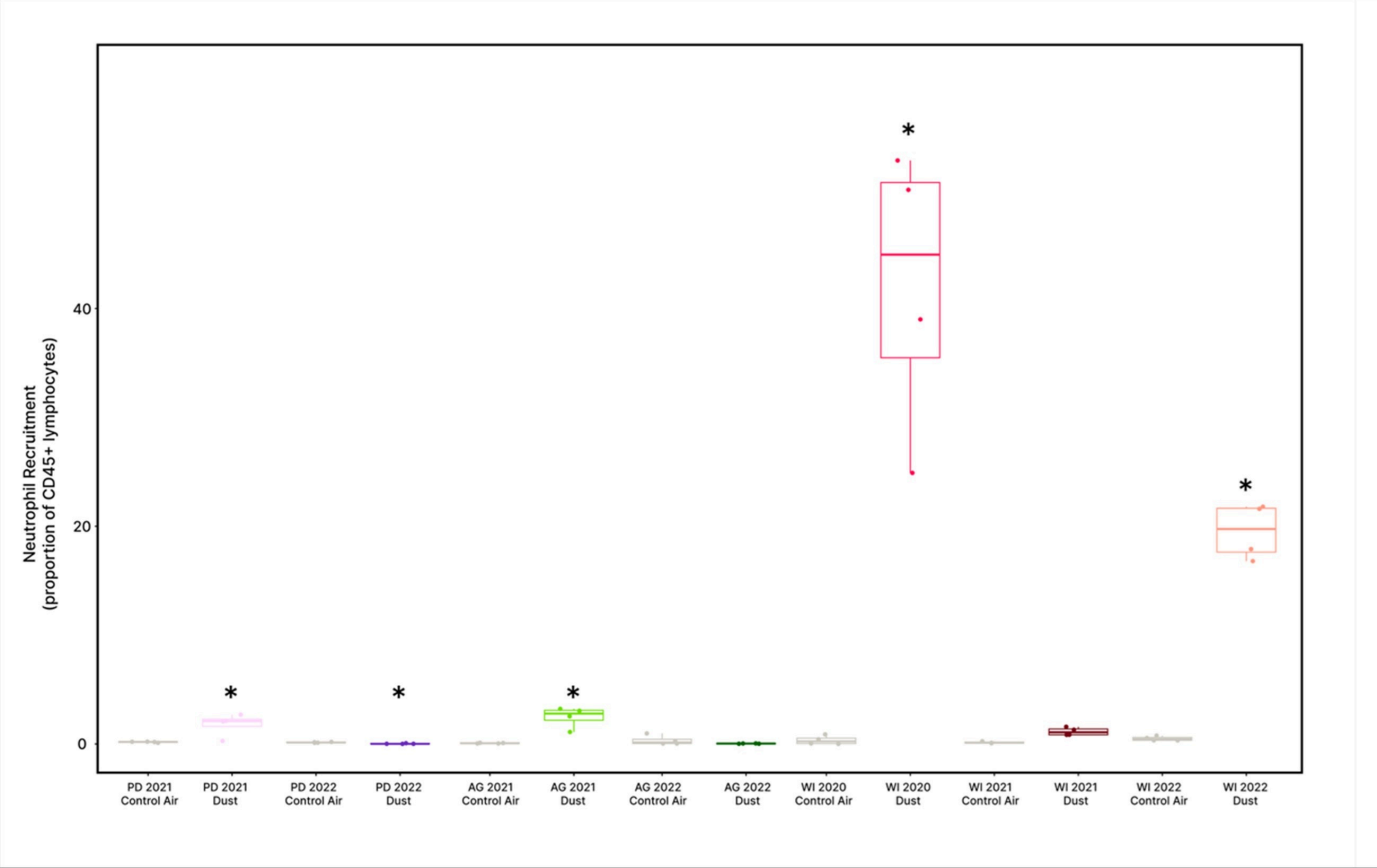
Lung neutrophil recruitment by treatment group. Neutrophil recruitment was analyzed as a proportion of CD45+ immune cells and pairwise comparisons were made between contemporaneous groups using a Wilcoxon signed rank test. Stars (*) indicate a significant pairwise comparison between a dust exposure group and its contemporaneous control (P<0.03). All pairs were significant with exception to the AG 2022 and WI 2021 exposures.

Host neutrophil recruitment was calculated as a proportion of CD54 + immune cells. The Wilcoxon signed rank test was used for pairwise comparisons between each dust-exposed group and their contemporaneous control air-exposed group. All pairwise comparisons were found to be equally significant according to the signed rank test (P = 0.030), except for WI 2021 dust-exposed mice and their respective control group (P = 0.060), and AG 2022 dust-exposed mice and their respective control group (P = 0.300). A box plot was used to visualize neutrophil recruitment (Fig. 9) among treatment groups and revealed that WI 2020 and WI 2022 dust-exposed mice experienced a greater magnitude of neutrophilic pulmonary inflammation in comparison to other treatment groups, despite the pairwise comparisons. Additionally, exposure to AG 2021 dust appeared to elicit a heighted neutrophilic response but to a lesser extent than Wister-exposed groups.

### Lipopolysaccharide (LPS) Concentrations in Salton Sea Dusts

LPS was quantified from normalized masses of dry dust samples collected at WI, AG, and PD during identical deployment dates in the fall of 2022 (Table. 1). It was determined that AG 2022 received the most dust mass over the collection period (2.9989 g/L), followed by PD 2022 (1.7253 g/L), then WI 2022 (1.5972 g/L). Despite this, WI 2022 had the highest endotoxin concentration (538.7534 EU/g), followed by AG 2022 (281.4738 EU/g) and PD 2022 (171.9529 EU/g).

### PM Surface Type Variation in Salton Sea Dust Samples

**Figure 10:**
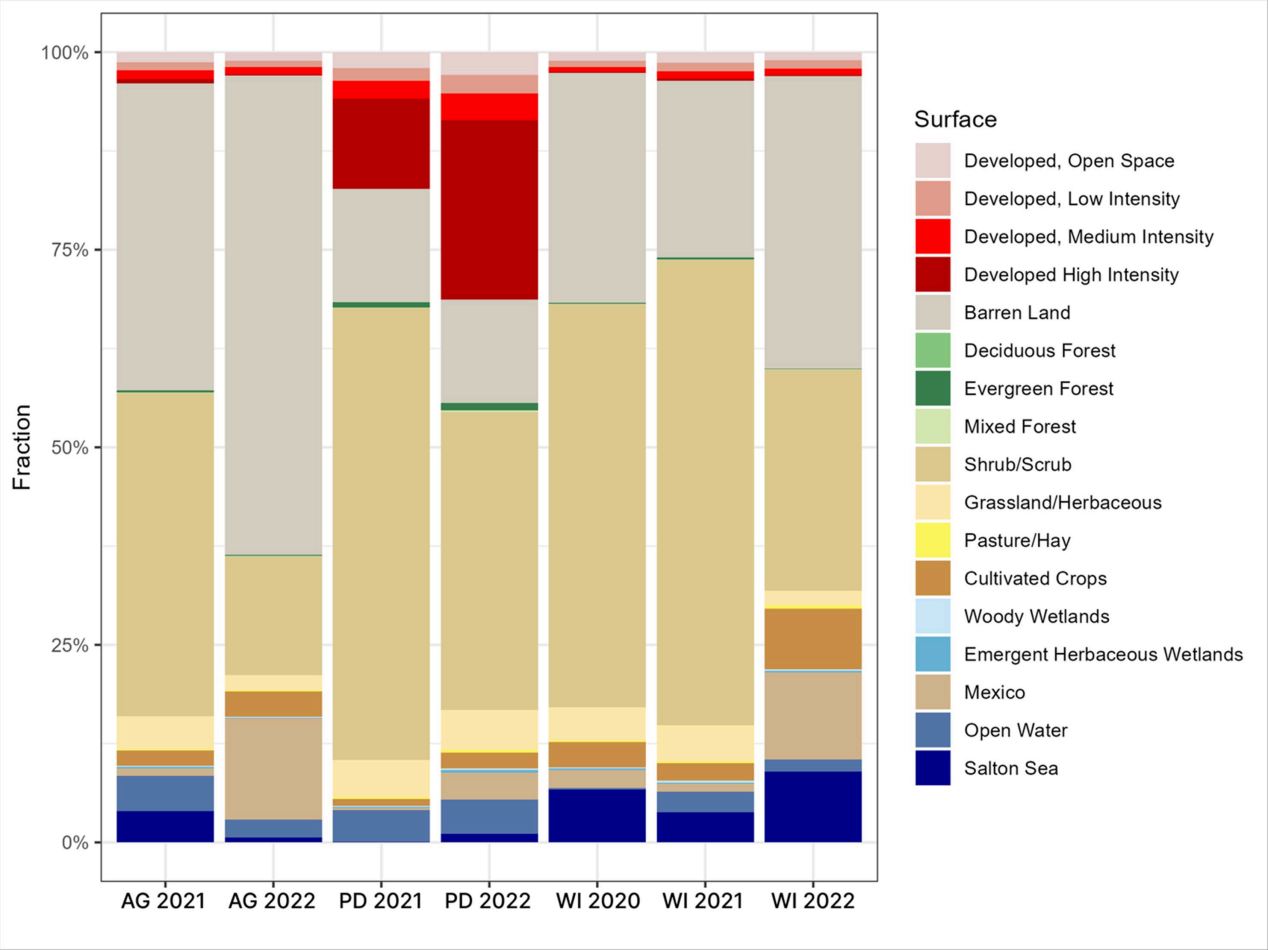
Lung neutrophil recruitment by treatment group. Neutrophil recruitment was analyzed as a proportion of CD45+ immune cells and pairwise comparisons were made between contemporaneous groups using a Wilcoxon signed rank test. Stars (*) indicate a significant pairwise comparison between a dust exposure group and its contemporaneous control (P<0.03). All pairs were significant with exception to the AG 2022 and WI 2021 exposures.

Back trajectory calculations were used to approximate frequencies of surface type contributions for the 2020, 2021, and 2022 dust collections utilized in the chamber exposure experiments (Fig. 10). It was observed that Wister consistently received higher fractions of Salton Sea dust contributions, regardless of collection year (WI 2020, 6.70%; WI 2021, 3.82%; WI 2022, 8.98%). In 2021, the Agricultural Site received comparable Salton Sea surface contributions as were received at WI (AG 2021, 3.97%), which contrasts with the frequency observed in 2022 (AG 2022, 0.60%). Palm Desert was observed to receive consistently low contributions from the Salton Sea surface (PD 2021, 0.08%; PD 2022, 1.10%).

## Discussion

We demonstrate that variability in dust and aerosol composition exerts a significant influence on both lung and fecal microbiomes, suggesting systemic host responses to environmental exposures beyond local pulmonary consequences. Following exposure to Salton Sea aerosols, neutrophilic pulmonary inflammation was significantly higher among mice exposed to aerosols collected close to the Sea (Fig. 9). These mice displayed tightly clustered and distinct lung microbiomes (Fig. 2), which were characterized by high taxa richness (Fig. 5) and low evenness (Fig. 3).

In the lung microbiomes of dust-exposed mice, we observed an enrichment of gram-negative taxa with wide metabolic capacities and opportunistic associations with human pathogenicity. This group included taxa such as *Acinetobacter*, which is clinically associated with pneumatic infection and natural-disaster-related disease outbreaks ^37^, and *Staphylococcus*, a genus associated with lung microbiomes of healthy and diseased patients ^38^. Another clinically relevant taxon, *Pseudomonas*, has been observed in both healthy and diseased clinical lavage samples ^39^, but is more commonly associated with lower airway inflammation and Chronic Obstructive Pulmonary Disease (COPD). Patients with enriched *Pseudomonas* have been shown to exhibit more frequent exacerbations and more severe disease symptoms ^40–42^.

Mouse fecal microbiomes were more diverse and compositionally unique when exposed to aerosols collected near the Sea (Fig. 7, SI Fig. 2). Previous research has pointed at the influence of environmental variables on the gut microbiome, particularly in the context of climate-related ecological shifts, public health burdens, and seasonal weather events ^21,43,44^. Broadly, psychosocial stress— which can be triggered by environmental burden— is consistently associated with gut microbiome dysbiosis, characterized by reduced diversity and compositional shifts ^45,46^. We expected that shifts in the fecal microbiome would be observed if chronic dust exposure and subsequent pulmonary inflammation had greater systemic stress effects on the host. In this case, the WI 2022 aerosol exposure had opposing effects on lung and fecal microbiome diversity, leading to increased lung and decreased fecal microbiome taxa richness and diversity.

Direct depletion of gut microbiome diversity has been associated with adverse health effects, as antibiotic-treated and germ-free mice have been shown to exhibit higher susceptibility to viral and bacterial respiratory infections. Further, the gut microbiome’s role in regulating systemic inflammatory responses has pointed to the “gut-lung axis” as a critical player in modulating COPD exacerbations and asthma via inflammatory mediators and ligand signaling through circulation or mucosa ^18,20,39^. Thus, significant depletion of fecal microbiome diversity in correlation with changes to lung microbiomes and pulmonary neutrophil recruitment suggest that chronic exposure to Salton Sea dust acts as a systemic stressor.

Despite all aerosols collected in 2022 eliciting a significant change in lung microbiome composition when compared to the control cohort, only mice exposed to WI 2022 experienced a high-magnitude neutrophilic inflammatory response and significant changes in fecal microbiome diversity in tandem. Although finer chemical analyses of these Salton Sea dusts were not thoroughly performed, back trajectory analyses were used to approximate surface type frequencies at each Salton Sea site for the exact duration of the collection period. These approximations reveal overall higher contributions from the Salton Sea’s surface at Wister, and especially during the 2022 collection period (Fig. 10). The biogeochemistry of the Salton Sea water column varies significantly by season due to organic matter cycling, as well as nutrient and oxygen availability, and thus leads to significant temporal variation in marine microbiome composition ^11,12^. This could likely extend to the playa surface and entrainment of novel assemblages of microorganisms in wind currents.

The nature of our methods assumes that no viable microbial cells were aerosolized in these chamber experiments, and we did not directly seed the lung microbiome through exposure. Yet it is possible that water-soluble bacterial byproducts remained in our aerosols at the time of exposure. One such microbial byproduct, lipopolysaccharide (LPS), is a bacterial endotoxin known to trigger and exacerbate pulmonary inflammation upon introduction ^47,48^. LPS is associated with both Salton Sea water and dust samples, and its concentration in the environment putatively contributes to regional pulmonary inflammation ^49^. We quantified LPS concentration in our 2022 aerosol samples as a proportion of the total dust mass obtained during that collection period. Wister 2022 displayed the highest concentration of LPS endotoxin, despite having the lowest mass of total dust collected (Table. 1).

Significant effects of host pulmonary inflammation and host microbiome diversity were observed following chronic exposure to aerosols collected in 2022 at Wister, a site displaying frequent contributions from the Salton Sea surface and high concentrations of LPS. We found that environmental conditions, reflected in local aerosol composition, independently affect host pulmonary health and microbiomes. In the case of the Salton Sea, our findings suggest that chronic exposure to aerosols around Wister, a site immediately southeast of the Salton Sea, triggers a systemic stress response in mice characterized by high pulmonary neutrophil recruitment, increased lung microbiome diversity, and decreased fecal microbiome diversity. Overall, our work demonstrates the variable capacity of environmental aerosol exposure to elicit health consequences relative to seasonal weather events.

## Supplementary Material

Supplementary Files

This is a list of supplementary files associated with this preprint. Click to download.

• TopacioMaltzMurineLungFecalDiversitySupp.docx

• floatimage1.png

## Figures and Tables

**Table 1.** LPS concentration in 2022 collections

Site	Dust collected (g/L)	LPS Concentration (EU/g)
PD 2022	1.7253	171.9529
AG 2022	2.9989	281.4737
WI 2022	1.5972	538.7534

## Data Availability

Lung and fecal microbiome sequencing data were deposited into the Sequence Read Archive database under BioProject ID PRJNA1399951 and are available at the following URL: https://www.ncbi.nlm.nih.gov/bioproject/1399951

## References

[1] Gilbert J. A. & Stephens B. Microbiology of the built environment. Nat. Rev. Microbiol. 16, 661–670 (2018). 30127345 10.1038/s41579-018-0065-5

[2] Johnston J. E., Razafy M., Lugo H., Olmedo L. & Farzan S. F. The disappearing Salton Sea: A critical reflection on the emerging environmental threat of disappearing saline lakes and potential impacts on children’s health. Sci. Total Environ. 663, 804–817 (2019). 30738261 10.1016/j.scitotenv.2019.01.365 PMC7232737

[3] Jones B. A. & Fleck J. Shrinking lakes, air pollution, and human health: Evidence from California’s Salton Sea. Sci. Total Environ. 712, 136490 (2020). 31931219 10.1016/j.scitotenv.2019.136490

[4] Calderón-Ezquerro M. D. C., Serrano-Silva N. & Brunner-Mendoza C. Aerobiological study of bacterial and fungal community composition in the atmosphere of Mexico City throughout an annual cycle. Environ. Pollut. 278, 116858 (2021). 33740598 10.1016/j.envpol.2021.116858

[5] Li H. Spatiotemporal variations of microbial assembly, interaction, and potential risk in urban dust. Environ. Int. 170, 107577 (2022). 36244231 10.1016/j.envint.2022.107577

[6] Maltz M. R. Landscape Topography and Regional Drought Alters Dust Microbiomes in the Sierra Nevada of California. Front. Microbiol. 13, 856454 (2022). 35836417 10.3389/fmicb.2022.856454 PMC9274194

[7] Freund H. Microbiome interactions and their ecological implications at the Salton Sea. Calif. Agric. 76, 16–26 (2022).

[8] Vogl R. A. & Henry R. N. Characteristics and contaminants of the Salton Sea sediments. in The Salton Sea (eds Barnum D. A., Elder J. F., Stephens D. & Friend M.) 47–54 (Springer Netherlands, Dordrecht, 2002). doi:10.1007/978-94-017-3459-2_3.

[9] Cohen M. J., Morrison J. I. & Glenn E. P. The Ecology and Future of the Salton Sea. (1999).

[10] Frie A. L., Dingle J. H., Ying S. C. & Bahreini R. The Effect of a Receding Saline Lake (The Salton Sea) on Airborne Particulate Matter Composition. Environ. Sci. Technol. 51, 8283–8292 (2017). 28697595 10.1021/acs.est.7b01773

[11] Hung C. Nutrient loading as a key cause of short- and long-term anthropogenic ecological degradation of the Salton Sea. Sci. Rep. 14, 31247 (2024). 39732884 10.1038/s41598-024-82633-y PMC11682391

[12] Freund L. Diversity of sulfur cycling halophiles within the Salton Sea, California’s largest lake. BMC Microbiol. 25, 120 (2025). 40045185 10.1186/s12866-025-03839-2 PMC11883979

[13] Freund L. Weather conditions structure the taxonomic and functional diversity of the aeolian dust microbiome. Front. Microbiol. 17, 1691133 (2026). 41960429 10.3389/fmicb.2026.1691133 PMC13057538

[14] Miao Y. Source-specific acute cardio-respiratory effects of ambient coarse particulate matter exposure in California’s Salton Sea region. Environ. Res. Health 3, 015006 (2025).

[15] Lin L. The airway microbiome mediates the interaction between environmental exposure and respiratory health in humans. Nat. Med. 29, 1750–1759 (2023). 37349537 10.1038/s41591-023-02424-2

[16] Biddle T. A. Aerosolized aqueous dust extracts collected near a drying lake trigger acute neutrophilic pulmonary inflammation reminiscent of microbial innate immune ligands. Sci. Total Environ. 858, 159882 (2023). 36334668 10.1016/j.scitotenv.2022.159882

[17] Maltz M. R. Lung microbiomes’ variable responses to dust exposure in mouse models of asthma. mSphere 10, e00209–25 (2025). 41117558 10.1128/msphere.00209-25 PMC12645928

[18] Wypych T. P., Wickramasinghe L. C. & Marsland B. J. The influence of the microbiome on respiratory health. Nat. Immunol. 20, 1279–1290 (2019). 31501577 10.1038/s41590-019-0451-9

[19] Perdijk O., Azzoni R. & Marsland B. J. The microbiome: an integral player in immune homeostasis and inflammation in the respiratory tract. Physiol. Rev. 104, 835–879 (2024). 38059886 10.1152/physrev.00020.2023

[20] McAleer J. P. & Kolls J. K. Contributions of the intestinal microbiome in lung immunity. Eur. J. Immunol. 48, 39–49 (2018). 28776643 10.1002/eji.201646721 PMC5762407

[21] Litchman E. Climate change effects on the human gut microbiome: complex mechanisms and global inequities. Lancet Planet. Health 9, e134–e144 (2025). 39986317 10.1016/S2542-5196(24)00332-2

[22] Aciego S. M. Dust outpaces bedrock in nutrient supply to montane forest ecosystems. Nat. Commun. 8, 14800 (2017). 28348371 10.1038/ncomms14800 PMC5379052

[23] Peng X. Establishment and characterization of a multi-purpose large animal exposure chamber for investigating health effects. Rev. Sci. Instrum. 90, 035115 (2019). 30927824 10.1063/1.5042097 PMC6910591

[24] Biddle T. A. Salton Sea aerosol exposure in mice induces a pulmonary response distinct from allergic inflammation. Sci. Total Environ. 792, 148450 (2021). 34157526 10.1016/j.scitotenv.2021.148450

[25] Lundberg D. S., Yourstone S., Mieczkowski P., Jones C. D. & Dangl J. L. Practical innovations for high-throughput amplicon sequencing. Nat. Methods 10, 999–1002 (2013). 23995388 10.1038/nmeth.2634

[26] Klindworth A. Evaluation of general 16S ribosomal RNA gene PCR primers for classical and next-generation sequencing-based diversity studies. Nucleic Acids Res. 41, e1–e1 (2013). 22933715 10.1093/nar/gks808 PMC3592464

[27] Andrews S. Andrews S. FastQC: A Quality Control Tool for High Throughput Sequence Data. 2010. https://www.bioinformatics.babraham.ac.uk/projects/fastqc/ (2010).

[28] Callahan B. J. DADA2: High-resolution sample inference from Illumina amplicon data. Nat. Methods 13, 581–583 (2016). 27214047 10.1038/nmeth.3869 PMC4927377

[29] Quast C. The SILVA ribosomal RNA gene database project: improved data processing and web-based tools. Nucleic Acids Res. 41, D590–D596 (2012). 23193283 10.1093/nar/gks1219 PMC3531112

[30] Davis N. M., Proctor D. M., Holmes S. P., Relman D. A. & Callahan B. J. Simple statistical identification and removal of contaminant sequences in marker-gene and metagenomics data. Microbiome 6, 226 (2018). 30558668 10.1186/s40168-018-0605-2 PMC6298009

[31] Lubbe S., Filzmoser P. & Templ M. Comparison of zero replacement strategies for compositional data with large numbers of zeros. Chemom. Intell. Lab. Syst. 210, 104248 (2021).

[32] Quinn T. P., Erb I., Richardson M. F. & Crowley T. M. Understanding sequencing data as compositions: an outlook and review. Bioinformatics 34, 2870–2878 (2018). 29608657 10.1093/bioinformatics/bty175 PMC6084572

[33] Quinn T. P. A field guide for the compositional analysis of any-omics data. GigaScience 8, giz107 (2019). 31544212 10.1093/gigascience/giz107 PMC6755255

[34] Martinez Arbizu P. pairwiseAdonis. https://github.com/pmartinezarbizu/pairwiseAdonis (2020).

[35] Stein A. F. NOAA’s HYSPLIT Atmospheric Transport and Dispersion Modeling System. Bull. Am. Meteorol. Soc. 96, 2059–2077 (2015).

[36] Lahti L. & Shetty S. Core microbiome. https://microbiome.github.io/tutorials/Core.html (2018).

[37] Munoz-Price L. S. & Weinstein R. A. Acinetobacter Infection. N. Engl. J. Med. 358, 1271–1281 (2008). 18354105 10.1056/NEJMra070741

[38] Marimón J. M. The Lung Microbiome in Health and Respiratory Diseases. Clin. Pulm. Med. 25, 131–137 (2018).

[39] He Y. Gut–lung axis: The microbial contributions and clinical implications. Crit. Rev. Microbiol. 43, 81–95 (2017). 27781554 10.1080/1040841X.2016.1176988

[40] Kostric M. Development of a Stable Lung Microbiome in Healthy Neonatal Mice. Microb. Ecol. 75, 529–542 (2018). 28905200 10.1007/s00248-017-1068-x

[41] Natalini J. G., Singh S. & Segal L. N. The dynamic lung microbiome in health and disease. Nat. Rev. Microbiol. 21, 222–235 (2023). 36385637 10.1038/s41579-022-00821-x PMC9668228

[42] Erb-Downward J. R. Analysis of the Lung Microbiome in the “Healthy” Smoker and in COPD. PLoS ONE 6, e16384 (2011). 21364979 10.1371/journal.pone.0016384 PMC3043049

[43] Davenport E. R. Seasonal Variation in Human Gut Microbiome Composition. PLoS ONE 9, e90731 (2014). 24618913 10.1371/journal.pone.0090731 PMC3949691

[44] Chakrabarti S. K. & Chattopadhyay D. Understanding The Eco-Gut Link: How Climate Shapes Gut Microbiome and Human Health. J. Community Med. Public Health Rep. https://doi.org/10.38207/JCMPHR/2024/AUG051105104 (2024) doi:10.38207/JCMPHR/2024/AUG051105104.

[45] Marwaha K. Exploring the complex relationship between psychosocial stress and the gut microbiome: implications for inflammation and immune modulation. J. Appl. Physiol. 138, 518–535 (2025). 39813028 10.1152/japplphysiol.00652.2024

[46] Bidell M. R., Hobbs A. L. V. & Lodise T. P. Gut microbiome health and dysbiosis: A clinical primer. Pharmacother. J. Hum. Pharmacol. Drug Ther. 42, 849–857 (2022). 10.1002/phar.2731 PMC9827978 36168753

[47] Liu X. Smoking related environmental microbes affecting the pulmonary microbiome in Chinese population. Sci. Total Environ. 829, 154652 (2022). 35307427 10.1016/j.scitotenv.2022.154652

[48] Poroyko V. Alterations of lung microbiota in a mouse model of LPS-induced lung injury. Am. J. Physiol.-Lung Cell. Mol. Physiol. 309, L76–L83 (2015). 25957290 10.1152/ajplung.00061.2014 PMC4491514

[49] Yisrael-Gayle K. Evidence for Aerosolized Environmental Bacterial Endotoxin as an Environmental Health Hazard. Preprint at 10.1101/2025.10.02.25337178 (In Review).

